# Cellular therapy with human autologous adipose-derived adult cells of stromal vascular fraction for alopecia areata

**DOI:** 10.1186/s13287-018-0889-y

**Published:** 2018-05-15

**Authors:** Rami Anderi, Nehman Makdissy, Albert Azar, Francine Rizk, Aline Hamade

**Affiliations:** 1 Cosmetic Plastic Surgery Center, Beirut, Lebanon; 2 Stem Cells, Organogenesis and and Regenerative Medicine, Lebanese University, Beirut, Lebanon; 3 Department of Biology, Faculty of Sciences 3, Lebanese University, Kobbe, Lebanon; 4 Reviva Regenerative Medicine Center, Middle East Institute of Health University Hospital, Bsalim, Lebanon; 5 Department of Biology, Laboratory of Therapeutic Innovation, Faculty of Sciences 2, Lebanese University, Fanar, Lebanon

**Keywords:** Baldness, Adipose-derived stromal vascular cells, Hair fall treatment, Stem cell, Mesenchymal stem cell, Hair loss, Alopecia

## Abstract

**Background:**

Most common forms of hair loss (alopecia) are caused by aberrant hair follicle cycling and changes in hair follicle morphology. However, current treatments for alopecia do not specifically target these processes. Adipose-derived stromal vascular cells (ADSVCs) that can be harvested from fat cells are one of the latest breakthroughs in the aesthetic field. The potential use of stem cell-based therapies (SCBT) for the repair and regeneration of various tissues and organs offers a paradigm shift that may provide alternative therapeutic solutions, which can be applied to prevent hair loss. This study aimed to present clinical cases of SCBT for the treatment of alopecia areata by transplantation of ADSVCs in the scalp.

**Methods:**

Twenty patients (9 women and 11 men) were recruited to our retrospectively registered study. After lipoaspiration, autologous ADSVCs were generated and characterized before the injection of 4–4.7 ×  10^6^ cells into the scalp of the patient. Hair regeneration was assessed by three clinical tests: the pull test, hair quality, and hair density.

**Results:**

All patients experienced hair regeneration, increased hair growth and decreased pull test 3 and 6 months after the treatment with ADSVCs [hair density (85.1 ± 8.7 vs 121.1 ± 12.5 hair/cm^2^, *P* < 0.0001), hair diameter (60.5 ± 1.8 vs 80.8 ± 2.4μ, *P* < 0.0001) and pull-test values (4.4 ± 0.3 vs 0.8 ± 0.2, *P* < 0.0001), untreated versus 6 months post-operative)]. Significant variation was observed between men and women only for hair diameter. No significant differences were observed with age.

**Conclusions:**

The obtained results prove the efficacy and the safety of the treatment, and satisfaction of the patients confirm the quality of the results.

## Background

Hair loss is one of the most common complaints among all patients consulting a dermatologist and is usually associated with severe psychological disturbances, distress, and symptoms of depression. Most common forms of hair loss (alopecia) are caused by aberrant hair follicle cycling and changes in hair follicle morphology. Cells with stem cell properties have recently been described in many integument appendages including feathers and teeth, but the hair follicle stands out as one of the best model systems for studying adult stem cells [1]. Hair follicles are accessible, well defined in terms of their developmental biology, and their stem cell populations are located in discrete compartments or niches [2–4]. Among several factors, alterations in hair follicle size may affect the hair loss: in fact, the size of a hair follicle is dependent of the volume of its dermal papilla which depends on the number of cells it contains [5].

There are three phases of hair growth, which every hair follicle undergoes. The first phase is when follicles undergo extremely rapid epithelial cell division and execute exquisitely timed differentiation programs when in the growing (Anagen) phase. The second phase is when follicles growth stops during a certain period, the (Catagen) phase [6, 7]. The third phase is when most follicles regress into structures that resemble immature developing follicles, after which they go into a period of mitotic quiescence, the (Telogen) phase. Stem cells are at the core of all hair dynamic events that includes a new burst of activity and further morphogenetic remodeling as the follicle grows again at the start of a new Anagen phase [8–10].

The term “stem cell” is given to a cell which has the ability to self-renew as well as to differentiate into defined cellular subtypes. Multipotent stem cells are present in different adult tissues such as bone marrow, dental pulp, adipose tissue, etc. [1]. Multipotent stem cells within adipose tissue [11], existing in adipose-derived stromal vascular cells (ADSVCs), are one of the most promising stem cell population identified, since human adipose tissue is easily obtained in large quantities with little patient discomfort and secretory factors from ADSVCs have been considered as a promising therapy for skin aging [12]. Therefore, the use of autologous ADSVCs can be promising for hair loss. Since the stromal vascular fraction (SVF) is saturated with stem cells among other cells derived from adipose tissue, cells can be called ADSVFC if they are used freshly, or ADSC/ADASC or others terms in cases of primary cells placed in culture having then the adherent feature, resulting in a set of mesenchymal stem cells (MSCs). In fact, contrary to cultured ADSCs, freshly isolated ADSVCs were shown to be highly positive for CD34, and positive for CD117 and HLA-DR. MSCs derived from adipose tissue when obtained by culture are mostly negative for CD34, and HLA-DR. This indicates clearly that primary cells are significantly more promising in case we need to maintain a certain level of CD34 in the graft.

In the present study, we aimed to use autologous ADSVCs graft for the treatment of alopecia areata and to assess the safety and effectiveness of the transplantation. The clinical trial of 20 patients shows the use of ADSVCs for hair growth and improvement as a valuable treatment.

## Methods

### Study subjects

White healthy subjects (*n* = 20, 38.3 ± 2.3 years, 9 women and 11 men) from the Middle East were enrolled in the study with no notable pathologic history in particular of hair diseases, with confirmed diagnostic of hair loss alopecia areata and all the selected subjects showed partial alopecia grade 1 or 2 at Ludwig Scale [10]. Subjects were excluded from this study if they had: histories of hair diseases other than hair loss/alopecia areata, conditions including moderate or severe head injury, burns, skin diseases, stroke, cerebral or bone damage particularly of the scalp or malignancies, brain abnormalities, learning disability, major medical or psychiatric illness in the previous 6 months, any metabolic/cardiovascular disease or evidence of cardiac/renal damage or malignancies, diabetes, hypertension, alcohol use, smoking, loss of weight during the last 2 years, chemotherapy, immunosuppressive, head/brain/abdominal surgeries, hormonal imbalances due to one or multiple factors (such as menopause, stress, depression, postpartum, chemotherapy, birth control, thyroid disorders, ovarian cysts, or others), medications known to cause hair loss (among others, medications for blood pressure, heart diseases, contraception, depression, etc.), disease and illness that may cause hair loss, chemical hair treatments, traction alopecia, compulsive hair pulling (Trichotillomania), poor nutrition, local infection (such as hepatitis, syphilis, herpes, HIV, etc.) or allergic reaction, abnormal physiologic levels of a comprehensive metabolic panel (CBC), vitamins, lipid and liver panel, hematocrit, hemoglobin, iron, ferritin, creatinine, coagulation factors, C-reactive protein, erythrocyte sedimentation rate, antinuclear antibodies, thyroid hormones, free and total testosterone, follicule-stimulating and luteinizing hormone. Treated alopecia areata cases were excluded; some example of treatments (steroid injections, corticosteroid creams and ointments, photochemotherapy, aromatherapy, acupuncture, herbal supplements, vitamins, platelet-rich plasma treatment, or any medications such as statins or other plasma cholesterol drug treatment, JAK-STAT pathway inhibitors, plaquenil, antihypertensive vasodilator medication (minoxidil), anthralin, immunomodulator therapy with squaric acid dibutylester (SADBE), diphenylcyclopropenone (DPCP) or the 5α-reductase inhibitor (finasteride), home remedies or others). Blood samples and abdominal fat lipoaspirates were collected from all the participants. No previous treatments were given to the patient before the procedure.

### Clinical tests

Three clinical tests, namely the pull test, hair quality, and hair density were performed, and pictures were obtained before and 3 and 6 months after the procedure to assure the authenticity of the results. The pictures were taken in the following positions to show the quantity of hair per square centimeter: from the front, of the parietal scalp, and close up [13–15].

### ADSVC preparation

The lipoaspirate [16] was diluted with sterile phosphate-buffered saline (PBS), Sigma-Aldrich, St. Louis, MO, USA) supplemented with antibiotics and centrifuged at 430 × g for 10 min (without brakes) to remove contaminating debris and red blood cells. The wash step was repeated 2–3 times depending on the quality of the specimen. The floating adipose tissue was digested with an equal volume of collagenase type I [10 mg/mL in PBS containing 5 mM Ca^2+^/Mg^2+^ (C0130, Sigma-Aldrich), final concentration 0.5%] at 37 °C for 30 min with shaking (250 rpm). The collagenase was inactivated by adding an equal volume of autologous serum, and the sample was centrifuged at 600 × g for 10 min. After centrifugation, the supernatant was discarded and the cell pellet was resuspended in NaCl 0.9% (Alpha Laboratories, Eastleigh, UK) and filtered through a 100 μm cell strainer (CS003 – PNC International Co. Ltd., Seoul, Korea) to remove debris. After centrifugation (300 RCF/5 min), 5 ml of the stromal vascular fraction were collected. All the processing must be realized within a maximal time of 90 min. The number of viable cells were determined manually (Trypan blue method) and validated on MACSQuant analyzer (Miltenyi-Biotech, Bergisch Gladbach, Germany) (7AAD staining method). All the quality control tests and injections were done with the obtained fresh samples.

### Assessments of cell immunophenotyping, viability, apoptosis, and telomerase activity

Freshly isolated cells were characterized for ADSVCs surface protein expression [17] by flow cytometry (MACSQuant analyzer, Miltenyi-Biotech) according to the manufacturer’s instructions. Cells were stained with the following antihuman-conjugated monoclonal antibodies: CD13-APC, CD14-PE, CD29-FITC, CD31-APC, CD34-PE, CD45-VioBlue, CD73-APC, CD90-FITC, CD105-VioBlue, CD144 (VE-Cadherin)-PE, CD146-Biotin, CD166-Biotin, HLA-ABC-FITC, HLA-DR-VioBlue, or relevant isotype-matched controls (Miltenyi-Biotech). Isotypes controls and automated compensation were settled to minimize false positive fluorescence and spectral overlap of fluorochromes respectively. Cell viability and apoptosis were assessed by the 7AAD/AnnexinV/PI assay. In fact, cell viability was first assessed manually using the Trypan blue (Sigma-Aldrich) exclusion assay, and the results were then validated by the 7AAD method by flow cytometry. The telomerase activity [18] was assessed by real-time qPCR (LightCycler 2.0, Roche, Basel, Switzerland) using the Quantitative Telomerase Detection Kit (Cat#MT3012, Allied Biotech, Taipei, Taiwan) according to the manufacturer’s instructions.

### ADSVCs injection and patient follow-up

The ADSVCs were injected into the scalp of the patient according to the following procedure: (1) the upper frontal, biparietal, and upper pyramidal area were first treated with the aseptic chlorhexidine without local anesthesia; (2) to reach the hair follicle area, the injection into the scalp area was performed with the following attributes: syringe, 1 cm^3^; needle, 4 mm; gauge, 30; and depth, 4 mm [19]; (3) 0.2 ml per injection was delivered perpendicularly, separated by 1 cm in a square shape all over the scalp marked previously; a total of 5 ml were injected in 25 spots; (4) after the injection was administered, the needle was kept in the scalp for 2 s. After the transplantation, the patient was prescribed nonsteroid anti-inflammatory and cephalexin antibiotics for 3 days. Patients were advised not to shower until 24 h after the procedure, not to sunbathe until after 1 week, and not to engage in sports until after 1 week; however, return to work can be on the same day. Follow-up for hair evaluationwas based on the hair cycles and was performed 1 week, 3 months, and 6 months after the procedure.

### Statistical analysis

For descriptive statistics, mean and variance were reported when appropriate. SPSS version 21.0 (IBM Corp., Armonk, NY, USA) was used for the statistical analysis. Comparisons between groups were performed using one-way ANOVA. Chi-square tests were applied to detect the difference in the rates between different groups. *P* values of less than 0.05 were considered significant.

## Results

In this study, the effect of hair regenerative ADSVC therapy was evaluated in 20 patients (9 females and 11 males) aged between 23 and 63 years old. First, based on a joint statement of the International Federation for Adipose Therapeutics and Science (IFATS) and the International Society for Cellular Therapy (ISCT) published in 2013, which point out the minimal phenotypic criteria to characterize the uncultured SVF population from adipose tissue [20], we characterized these freshly isolated cells. In fact, the immunophenotyping of the transplanted cells showed clearly a heterogeneous population of freshly isolated cells, which expressed not only the mesenchymal stem cells markers, but also the panhematopoietic/monocyte/macrophage/endothelial/pericyte markers and particularly high levels of CD34. These cells strongly expressed HLA-ABC but weakly expressed HLA-DR markers (Table 1). Cell viability, as assessed by Trypan blue and validated by 7AAD staining, was > 96% and cells affected by an early apoptosis were rare (Table 1); it is important to note that the total time of processing was less than 120 min, and a prolonged processing time affected the viability of the cells. That is why, we managed the processing of all samples during a maximal time of 120 min and transplanted the cells in a total time not exceeding 3 h. A significant decrease in the viability was observed after 4–6 h (8%, 24%, and 31% of decrement in cell viability rate after 4, 5, and 6 h, respectively) (data not shown). On the other hand, a total number of 4 to 4.7 × 10^6^ cells were transplanted: in fact, 0.2 mL containing 0.160–0.188 × 10^6^ cells were injected per spot (total = 25 spots, 5 mL). In agreement with Varma et al. [21], our results indicated that freshly isolated ADSVCs cells were shown to be highly positive for CD34, contrary to the expression of CD105 and especially CD166 which were relatively low (3.19% and 6.37%, respectively) (Table 1): to maintain a minimum level of these cells in the sample, this led us to consider at least the presence of 5000 CD105^+^/CD166^+^ cells in the 0.2 mL of transplanted sample per spot of injection, which prompted us to choose the minimum concentration of 160,000 cells/spot of injection (= 4 × 10^6^ total cells/25 spots/per subject). Importantly, to avoid any aggregation of the cells, which was observed in cases where the cell concentration was > 200,000 cells per 0.2 mL, and to maintain the minimum levels of CD105^+^ and CD166^+^ cells per injection, a total of 4.0–4.7 × 10^6^ cells was delivered to the subjects.

**Table 1:** Immunophenotyping and apoptotic index of cell surface markers expressed by total nucleated SVF cells

ADSVCs (SVF cells) Freshly isolated from adipose tissue	% of total cells
CD13^(+)^	71.06 ± 5.08
CD14^(+)^	10.28 ± 4.11
CD29^(+)^	32.44 ± 2.09
CD31^(+)^	18.56 ± 2.83
CD34^(+)^	71.27 ± 3.54
CD45^(+)^	27.70 ± 6.33
CD73^(+)^	27.07 ± 5.81
CD90^(+)^	55.27 ± 4.16
CD105^(+)^	6.37 ± 4.12
CD144 (VE cadherin)^(+)^	18.62 ± 4.92
CD146^(+)^	24.45 ± 2.17
CD166^(+)^	3.19 ± 2.05
HLA-ABC^(+)^	98.04 ± 1.12
HLA-DR^(+)^	10.17 ± 3.62
Annexin V^(−)^/PI^(−)^/7AAD^(−)^ (viable cells)	98.97 ± 0.71
Annexin V^(+)^/PI^(−)^/7AAD^(−)^ (early-apoptotic cells)	1.02 ± 0.54
Annexin V^(+)^/PI^(+)^/7AAD^(+)^ (late apoptotic, necrotic cells)	0.17 ± 0.82

2 × 10^5^ cells were labeled with fluorescence-coupled antibodies against the indicated cell surface markers, Annexin V, 7AAD, propidium iodide solution (PI), and analyzed using a MACSQuant flow analyzer as indicated in Materials and Methods. The results are expressed as the mean ± SEM of all subjects (*n* = 20) each performed in duplicate

*ADSVC* adipose-derived stromal vascular cell, *SVF* stromal vascular fraction

Second, we assessed the hair loss and growth which were determined as changes in hair density (n/cm^2^) and hair diameter (μ), as well as the pull test (Table 2). Overall, 55% of the patients showed medium diameter hair and 45% showed fine hair. All the study subjects showed abnormal hair density (density < 175 hair/ cm^2^ in 100% of the subjects). All the patients showed a value superior to 0 for the pull test. In addition, no significant variations were observed with age.

**Table 2:** Patient’s profile before ADSVCs injection

Patient’s initials	Age, yrs	Gender, M/F	Diameter preoperatively (μ)	Density preoperatively (h/cm^2^)	Pull test	Number of cells × 10^6^ injected/ 25 spots	Cell viability (%)	Apoptotic index (%)	Telomerase test
NM	23	F	72	72	5	4.20	99.51	0.32	Negative
AR	25	F	67	37	4	4.31	96.32	3.21	Negative
CK	27	F	72	98	2	4.32	99.02	1.00	Negative
ZS	37	F	66	134	3	4.19	99.14	0.98	Negative
RC	38	F	63	65	3	4.30	98.27	1.62	Negative
WK	43	F	64	43	4	4.49	99.82	0.44	Negative
GC	54	F	70	143	6	4.08	99.05	0.36	Negative
AM	56	F	60	30	4	4.70	96.42	3.11	Negative
MM	63	F	68	160	6	4.60	99.55	0.03	Negative
FR	27	M	65	66	2	4.58	98.28	1.09	Negative
MB	32	M	50	34	3	4.35	99.11	0.45	Negative
RO	32	M	48	127	5	4.22	99.73	0.16	Negative
SF	32	M	63	157	3	4.03	99.56	0.33	Negative
IA	34	M	57	113	5	4.46	99.70	0.37	Negative
SH	36	M	56	97	6	4.01	99.52	0.44	Negative
PC	37	M	63	65	4	4.50	99.58	0.61	Negative
HB	38	M	54	101	5	4.70	99.63	0.55	Negative
TB	38	M	56	88	8	4.22	99.59	0.11	Negative
OC	43	M	56	76	4	4.07	99.00	0.28	Negative
MH	51	M	41	68	5	4.26	99.81	0.24	Negative

Diameter: fine hair ≤ 60 μ; medium hair 60 to 80 μ, good hair ≥ 80 μ; Density: quantity of hair per cm^2^, normal is 175 to 300 hair/cm^2^; Pull test is the quantity of hair pulled with the finger pull test, normal is zero. 0.2 ml per spot of injection was delivered perpendicularly, separated by 1 cm in a square shape all over the scalp and a total of 5 ml were injected in 25 spots. Cell viability and apoptotic index as assessed by labeled cells with Annexin V/propidium iodide (PI)/7AAD, are expressed respectively as the percentage of Annexin V^(−)^/PI^(−)^/7AAD^(−)^ or Annexin V^(+)^ cells divided by total cells

*ADSVCs* adipose-derived stromal vascular cells

### ADSVCs injection increases hair diameter

**Fig. 1:**
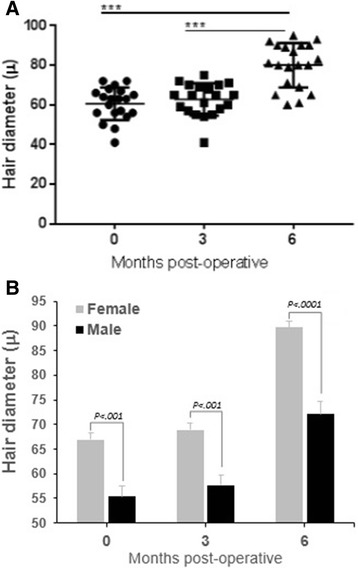
Difference in hair diameter between patients before and 3 or 6 months after ADSVCs transplantation. **a** Whole population. **b** Variation between male and female. ^***^P < 0.0001

Our results are based on the density of hair per square centimeter, the diameter of the hair, and the pull test performed manually by the same physician in all phases. Table 3 shows the results obtained at 3 and 6 months after the injection of ADSVCs in comparison to the preoperative ones. As shown in Table 2, the hair diameter increased significantly (*P* < 0.0001) with the injection of ADSVCs, especially 6 months after the treatment (80.8 ± 2.4 μ and 62.8 ± 1.7 μ vs. 60.5 ± 1.8 μ for 6 and 3 months postoperatively vs. preoperatively) (Fig. 1a). In total, 19 out of 20 patients showed improved hair diameter; only one patient did not show any improvement. Approximately, on average, 32% improvement was obtained, and maximum improvement was approximately 50.1%. Significant variation was observed between males and females (Fig. 1b).

**Table 3:** Comparison of hair diameter before (preoperative), 3 and 6 months (postoperative) after ADSVCs treatment

Patient’s initials	Age, yrs	Gender, M/F	Diameter Preoperatively (μ)	Diameter 3 months postoperatively (μ)	Diameter 6 months postoperatively (μ)
NM	23	F	72	71	93
AR	25	F	67	70	87
CK	27	F	72	75	92
ZS	37	F	66	68	89
RC	38	F	63	70	81
WK	43	F	64	65	90
GC	54	F	70	72	95
AM	56	F	60	60	90
MM	63	F	68	69	90
FR	27	M	65	65	65
MB	32	M	50	55	65
RO	32	M	48	55	61
SF	32	M	63	65	83
IA	34	M	57	57	79
SH	36	M	56	59	80
PC	37	M	63	65	80
HB	38	M	54	54	69
TB	38	M	56	61	80
OC	43	M	56	58	71
MH	51	M	41	41	60

Diameter; fine hair ≤ 60 μ ; medium hair 60 to 80 μ , thick hair ≥ 80 μ

*ADSVCs* adipose-derived stromal vascular cells

### ADSVCs injection improves hair density

**Fig. 2:**
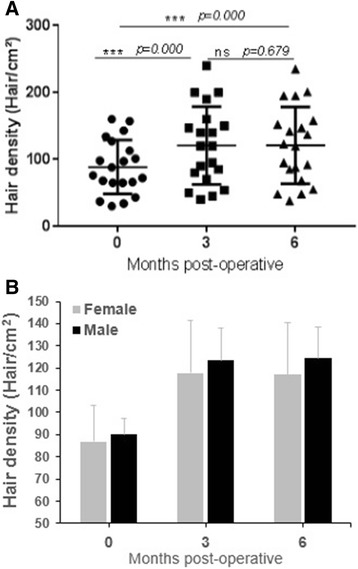
Difference in hair density between patients before and 3 or 6 months after ADSVCs transplantation. **a** Whole population. **b** Variation between male and female. ^***^P < 0.0001; ns, non-significant

We can note that the hair density was significantly (*P* < 0.0001) augmented after treatment with ADSVCs (121.1 ± 12.5 and 120.8 ± 12.6 vs. 85.1 ± 8.7 for 6 and 3 months postoperatively vs. preoperatively) (Table 4). The mean growth was approximately 36%, and the optimal effect was 61.2% (Fig. 2a). The hair growth occurred during the first phase. Of the 20 patients studied, only two did not show any significant improvement. No significant differences were observed between males and females (Fig. 2b).

**Table 4:** Comparison of hair density before (preoperative), 3 and 6 months (postoperative) after ADSVCs treatment

Patient’s initials	Age, yrs	Gender, M/F	Density Preoperative (hair/cm^2^)	Density 3 months postoperative (hair/cm^2^)	Density 6 months postoperative (hair/cm^2^)
NM	23	F	72	95	92
AR	25	F	37	40	38
CK	27	F	98	140	142
ZS	37	F	134	160	158
RC	38	F	65	80	85
WK	43	F	43	54	55
GC	54	F	143	200	201
AM	56	F	30	50	48
MM	63	F	160	240	235
FR	27	M	66	70	68
MB	32	M	34	45	48
RO	32	M	127	190	195
SF	32	M	157	200	195
IA	34	M	113	147	147
SH	36	M	97	120	123
PC	37	M	65	85	88
HB	38	M	101	150	152
TB	38	M	88	140	137
OC	43	M	76	120	120
MH	51	M	68	90	95

Hair density or trichometry is the quantity of hair per cm^2^, normal is 175 to 300 hair/cm^2^

*ADSVCs* adipose-derived stromal vascular cells

### ADSVC injection increases pull test

**Fig. 3:**
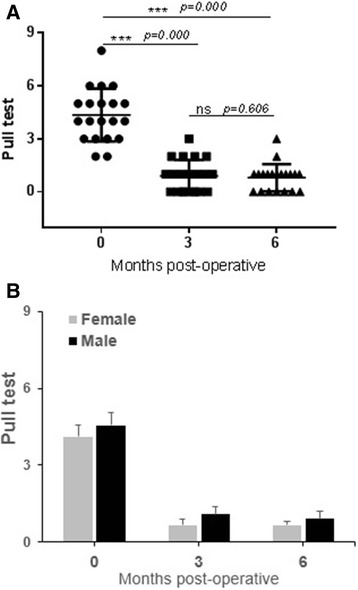
Comparison of pull test results before and after 3 months and 6 months after ADSVCs transplantation. **a** Whole population. **b** Variation between male and female. ^***^P < 0.0001; ns, non-significant

The results of the pull test showed a significant decrease in the number of extracted hair (*P* < 0.0001) following ADSVC treatments (0.80 ± 0.17 and 0.90 ± 0.20 vs. 4.35 ± 0.33 for 6 and 3 months postoperatively vs. preoperatively). We could notice that the hair became stronger at 3 and 6 months postoperatively, leading to values of mainly 0 and 1 in the pull test (Table 5, Fig. 3a). In fact, the values of the pull test in the control group ranged between 2 and 8; however, they were markedly inferior in the ADSVC-treated group. In total, 2 of the 20 patients showed no significant improvements. All other patients had normal responses. In addition, no significant differences were observed between males and females (Fig. 3b).

**Table 5:** Comparison of hair pull test before (preoperative), 3 and 6 months (postoperative) after ADSVCs treatment

Patient’s initials	Age, yrs	Gender, M/F	Density preoperative (hair/cm^2^)	Density 3 months postoperative (hair/cm^2^)	Density 6 months postoperative (hair/cm^2^)
NM	23	F	5	0	1
AR	25	F	4	1	1
CK	27	F	2	0	0
ZS	37	F	3	0	0
RC	38	F	3	0	1
WK	43	F	4	1	1
GC	54	F	6	2	1
AM	56	F	4	1	0
MM	63	F	6	1	1
FR	27	M	2	0	0
MB	32	M	3	0	0
RO	32	M	5	2	2
SF	32	M	3	0	0
IA	34	M	5	1	1
SH	36	M	6	2	0
PC	37	M	4	1	1
HB	38	M	5	1	1
TB	38	M	8	2	3
OC	43	M	4	0	1
MH	51	M	5	3	1

The pull test is by pulling the hair, the number of extracted pulled hairs must be between 0 and 1 in normal patients

*ADSVCs* adipose-derived stromal vascular cells

## Discussion

Hair fall treatment is a challenge for many doctors; however, hair fall treatment can be performed in multiple ways. To date, no studies have shown the hair fall treatment can be performed using ADSVCs with satisfactory results. Our research is unique and the first in the field of hair fall treatment to use ADSVCs. Most of the studies conducted in the past were based on hematopoietically derived plasma or ADSVC-conditioned medium (ADSVC-CM).

**Fig. 4:**
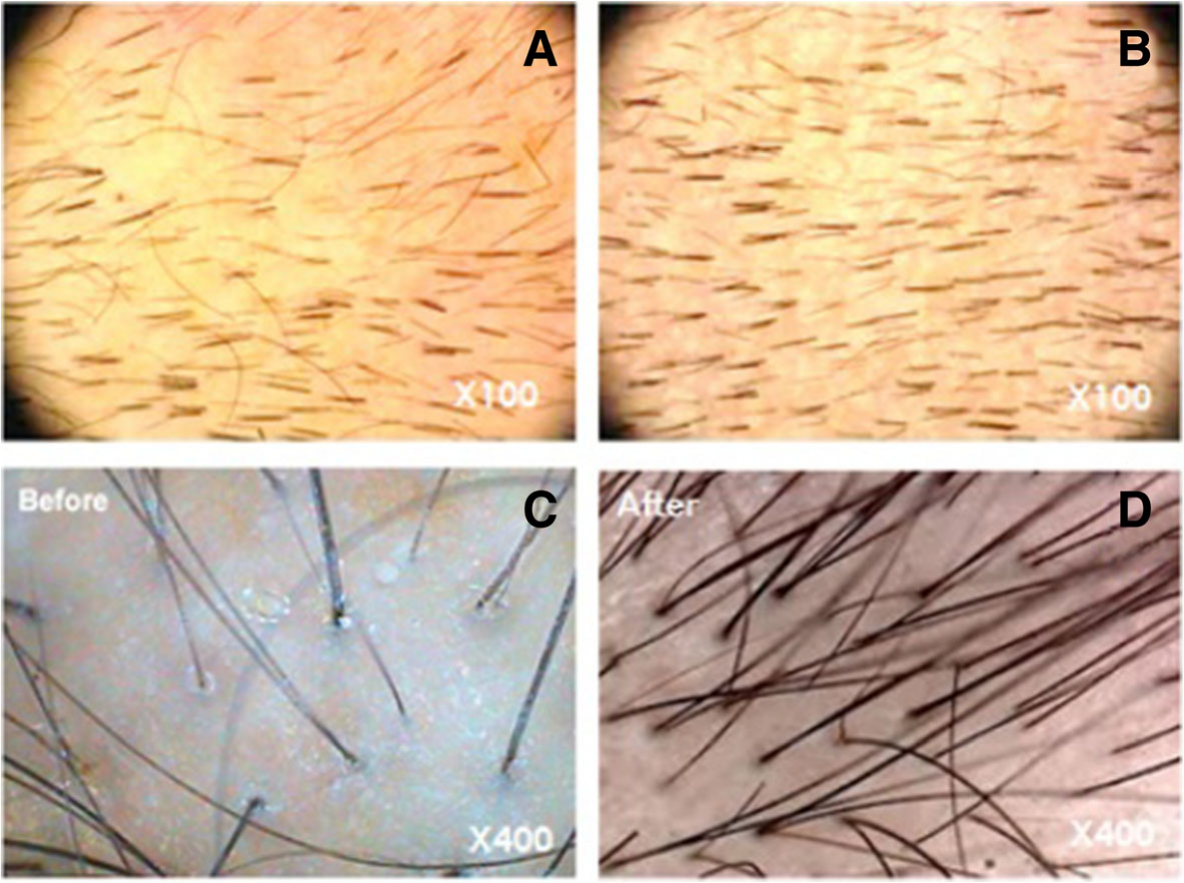
Photographs documenting the increase in hair counts after adipose-derived stem cell therapy*.* Representative images of the scalp before (**a-c**) and 6 months after ADSVCs transplantation (**b-d**)

This study showed that the transplantation of autologous ADSVCs is safe and effective and can be considered an encouraging cell-based therapy for the treatment of alopecia and a nonsurgical hair loss treatment. Hair growth and thickness were markedly improved 6 months after the treatment (Fig. 4).

Reports of the use of stem cells in the treatment of alopecia are rare. Researchers have utilized bone marrow/cord blood-derived stem cells or ADSVC-CM. However, to our knowledge, there is no study that investigated the effect of ADSVCs for the treatment of alopecia. Our results on the characterization of the isolated and transplanted cells agreed with the previously described immunophenotype [17, 21, 22]. Li et al. [23] recently showed that patients with severe alopecia areata showed improved hair regrowth and quality of life after receiving stem cell educator therapy. They demonstrated the safety and efficacy of a new method where the mononuclear cells are separated from the whole blood and were allowed to briefly interact with adherent human cord blood-derived multipotent stem cells, and the “educated” autologous cells were returned to the patient’s circulation. Fukuoka et al. [24] showed that treatment with ADSVC-CM effectively activated hair regeneration; ADSVC-CM is rich in growth factors such as vascular endothelial growth factor, hepatocyte growth factor, platelet-derived growth factor, and insulin-like growth factor 1. Another study with a female pattern hair loss treated with ADSVC-CM showed that the treatment increased the hair density and thickness [25]. Recently, it was reported that autologous bone marrow-derived mononuclear cells seem to be a safe, tolerable, and effective treatment for the management of both resistant alopecia areata and androgenetic alopecia [26].

The injected ADSVCs in this study might release growth factors, thus promoting vascularization, encouraging new capillaries to form, increasing the production of hair, and improving the supply of blood to the scalp. This provides an ideal environment for the hair follicles to grow new, denser, and healthy hair. This research is the beginning of many series of studies that will be conducted in the future to improve the results and decrease the cost of the procedure to make it more efficient and affordable to patients. It is important to note that most cases of alopecia areata (approximately 80%) resolve spontaneously especially first cases of alopecia areata. It will be interesting to study the efficacy of ADSVCs transplantation for grade III and advanced cases of alopecia areata.

## Conclusions

In conclusion, treatment using ADSVCs appears highly effective for alopecia areata and may represent a new avenue of therapy for hair regeneration. ADSVC injection promotes good stability of the hair by increasing the hair density, the hair diameter, and decreasing the pull test to almost zero. Furthermore, patients must be very well selected depending on their lifestyle, the cause of hair fall and baldness grade to obtain a good result with this procedure.

## Abbreviations

ADASC: Adipose-derived adult stem cell
ADSC: Adipose-derived stem cell
ADMSC: Adipose-derived-mesenchymal stem cell
ADSVC: Adipose-derived stromal vascular cell
ADSVC-CM: ADSVC-conditioned medium
CD: Cluster of differentiation
IFATS: International Federation for Adipose Therapeutics and Science
ISCT: International Society for Cellular Therapy
MSC: Mesenchymal stem cell
PBS: Phosphate-buffered saline
SCBT: Stem cell-based therapies
SVF: Stromal vascular fraction
